# Migraine and functional neurological disorder (FND)—a review of comorbidity and potential overlap

**DOI:** 10.1093/braincomms/fcaf288

**Published:** 2025-08-04

**Authors:** Jon Stone, Jan Coebergh, Lujain Khoja, Matthew Butler, Timothy R Nicholson, David W Dodick

**Affiliations:** Centre for Clinical Brain Sciences, University of Edinburgh, Edinburgh EH16 4SB, UK; Department of Neurology, St. George’s Hospital, London, UK; Faculty of Medicine, University of Jeddah, Jeddah, Saudi Arabia; Neuropsychiatry Research and Education Group, Institute of Psychiatry, Psychology and Neuroscience, King’s College London, London, UK; Neuropsychiatry Research and Education Group, Institute of Psychiatry, Psychology and Neuroscience, King’s College London, London, UK; Neuropsychiatry Research and Education Group, Institute of Psychiatry, Psychology and Neuroscience, King’s College London, London, UK; Department of Neurology, Mayo Clinic, Phoenix, AZ, USA; Atria Academy of Science and Medicine, New York, NY, USA

**Keywords:** migraine, functional neurological disorder, conversion disorder, epidemiology, comorbidity

## Abstract

Migraine and functional neurological disorder (FND) are two of the most common conditions in neurological practice. It is assumed that the two conditions have distinct underlying mechanisms. However, it can be clinically challenging to disentangle their relative contributions to a patient's symptoms. In addition, apart from the relationship between persistent postural perceptual dizziness (PPPD) and migraine, the frequency of co-occurrence has not been characterized in detail. Contemporary conceptualizations of FND have driven a re-evaluation of its relationship to other neurological disorders, including migraine. We carried out a narrative review of the literature examining the co-occurrence of migraine and FND. We also explored their comorbidities, aetiological risk factors and mechanisms, focusing especially on areas of potential overlap.

Our review suggests increased frequency of migraine in people with functional seizures compared to epilepsy, but data from people with functional motor symptoms is mixed. Robust epidemiological studies evaluating the frequency of FND in migraine are lacking. Similar to other neurological disorders, migraine is an established trigger of FND. Female gender, adverse childhood experiences and comorbid psychiatric and functional disorders, such as irritable bowel syndrome and fibromyalgia, are more common in both conditions than in controls, but perhaps more so in FND. Mechanistic research in both conditions highlights converging frameworks of dysregulated allostatic/stress responses in the context of predictive processing models of the brain. This has implications for pharmaceutical and rehabilitation treatments.

The relationship between migraine and FND is poorly studied. An overview of their overlap offers a model of non-dualistic thinking within a clinical neuroscience framework for future studies.

## Teaser

Migraine and functional neurological disorder (FND) are two of the most common conditions in neurological practice. Stone *et al*. review the existing literature looking at the clinical, epidemiological and mechanistic overlap of the two conditions. Despite limited literature, there are important treatment implications for studying the overlap of these disorders.

## Graphical Abstract

**Graphical Abstract:**
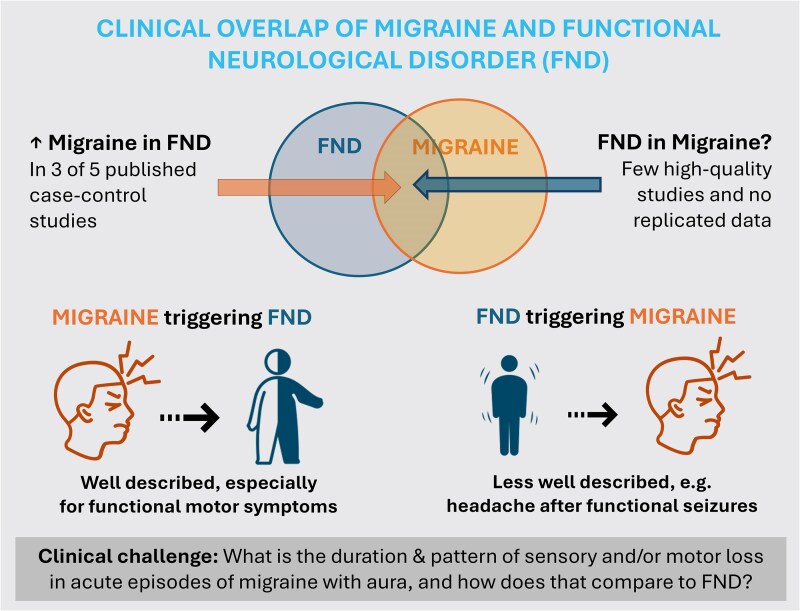

## Introduction

Migraine and functional neurological disorder (FND) are two of the most common conditions seen in neurological practice.^1^ Both conditions significantly impair occupational and social functioning in working-age adults. Both are clinical diagnoses and can be conceptualized as arising, in broad terms, primarily from disturbance of brain function rather than structural changes to the nervous system, although this is also true of most psychiatric disorders as well as some other neurological conditions (e.g. some epilepsies, ataxias and dystonias) and others that sit in between those categories such as Tourette syndrome.^2,3^ Both conditions can lead to a wide variety of neurological symptoms, which may be mistaken for one another, and they may also co-exist.

Major research efforts in the last few decades have increased the mechanistic understanding of migraine and have led to the linked development of effective treatment options.^2^ FND has only recently received significant research attention; hence, mechanistic understanding and evidence-based treatments are less developed.^3^ Excepting the overlap between persistent postural perceptual dizziness and migraine,^4,5^ there has been little conceptual or empirical research exploring how these two disorders relate to each other, despite commonly encountered clinical challenges in determining whether one or both are present and their relative contributions to different symptoms.^6,7^

In this review, we examine and discuss the intersection between FND and migraine, informed by a review of the research into the frequency of their co-occurrence. We review the clinical similarities and differences between these disorders, shared risk factors and comorbidities and how both can co-exist and potentially interact. We explore some possible shared pathophysiological mechanisms and their implications for management and propose a future research agenda to investigate the relationship between these two common disorders with major health and socio-economic impacts.

**Figure 1:**
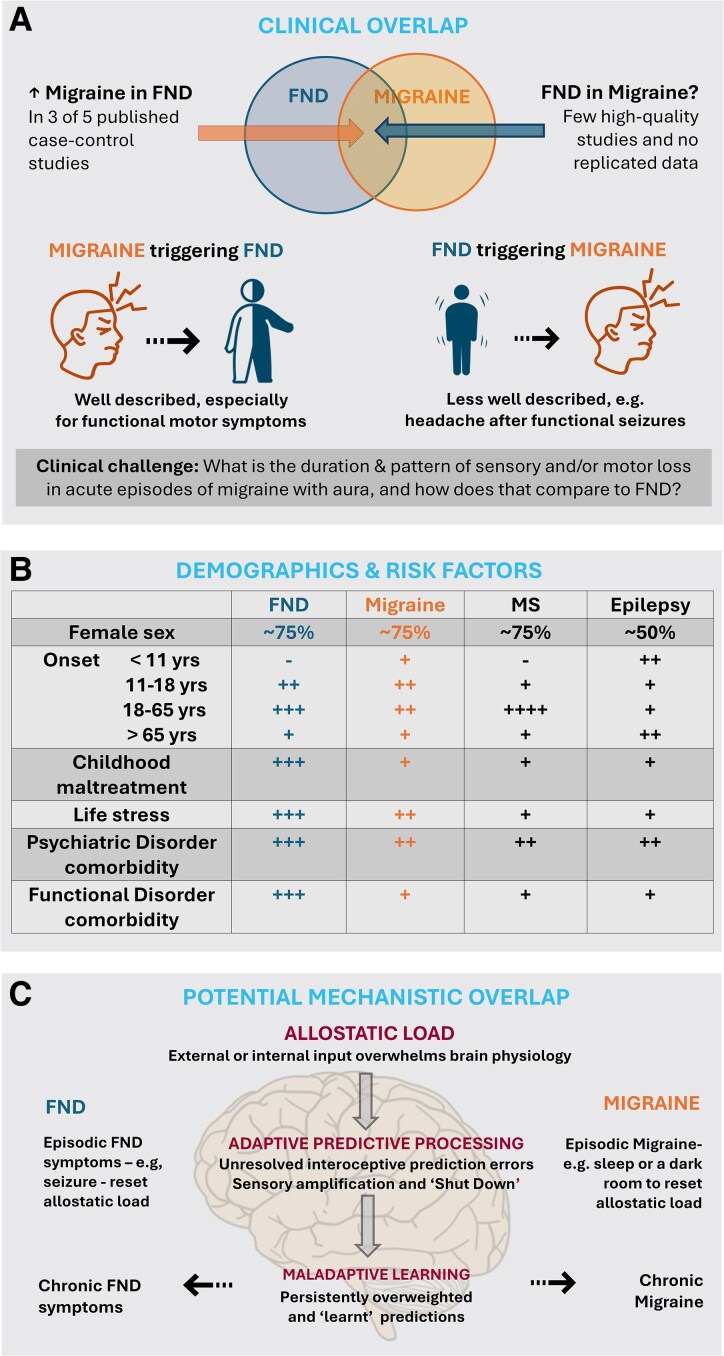
Migraine and FND—a visual summary of the review. (**A**) Clinical overlap. (**B**) Demographics and risk factors. (**C**) Potential mechanistic overlap. FND, functional neurological disorder; MS, multiple sclerosis.

Due to the size of the two fields, we considered systematic review methodology to be unwieldy for the purpose of this article. Instead, we undertook a comprehensive review of the literature (Box 1), paying particular attention to the largest epidemiological studies and other systematic reviews on topics relevant to shared epidemiology and pathophysiological mechanisms relating to migraine and FND. Where relevant for context, we also included literature related to other common neurological conditions such as epilepsy and multiple sclerosis. An infographic summarizing the review is shown in Fig. 1.

Box 1 Search methods We searched the databases PubMed and PsycINFO and the reference lists of eligible studies from inception to March 2024. The following search terms were used: ‘migraine’; ‘functional neurological disorder’; ‘conversion disorder’; ‘functional movement disorder’; ‘psychogenic movement disorder’; ‘psychogenic’, ‘dissociative’ or ‘nonepileptic’; and ‘seizure’, ‘attack’ or ‘spell’. We did not review studies of persistent postural perceptual dizziness or its overlap with vestibular migraine, since this has been discussed elsewhere, and because of the lack of a review of the overlap of FND with more standard motor and seizure subtypes.^4,5^ There were no language restrictions. The final reference list was generated on the basis of relevance to the headings covered in this review and supplemented with an additional updated search in March 2025.

### History

Both migraine and FND have been described in the medical literature since antiquity.^8,9^ Despite this, discussion of their overlap and potential relationship is relatively sparse. The influential neurologist William Gowers (1845–1915) wrote extensively about hysteria and migraine in his 1890 manual, but did not mention any overlap between the two conditions, despite a discussion of the overlap between migraine and epilepsy.^10^ Similarly, the physician and pioneering psychologist Pierre Janet (1859–1947) did not make any reference to migraine or headache in his seminal work on hysteria.^11^

However, there are some notable exceptions with some key leaders in clinical neuroscience discussing the overlap and potential relationship. For example, in his 1856 treatise on 430 women with ‘hysteria’, the physician Pierre Briquet (1796–1881) found headache in 300 of 356 patients, concluding that ‘*headache is one of the distortions of sensations most frequently found in hysterical women ….*’ noting a particular overlap with stammering, dizziness and mental confusion.^12^ Sigmund Freud (1856–1939), whose influence dominated the field of hysteria in the 20th century, wrote in his description of Frau Emmy von N in *Studies in Hysteria* that ‘*As we know, many neuropathic women very often have hysterical attacks (spasms and deliria) along with an attack of migrain*’.^13^

In 1890, Joseph Babinski described four cases of ‘*migraine ophthalmique hystérique*’^14^ in which migraine was associated with episodes of impaired awareness with features of FND, including eyelid fluttering or modulation by suggestion. Oppenheim, another leading neurologist of his time, wrote in his 1911 textbook that ‘*I have noticed the transformation of paroxysmal migraine in to the chronic form specially in neurasthenic or hysterical patients*’.^15^ In 1948, Barraquer-Ferré, noted ‘*several histories of individuals who displayed hysterical or emotionally pathological manifestations and so on, as a part of the cycle of migraine attacks. When this state persists, patients may now and then exhibit signs of hysterical dystonia, in all cases transitory*’.^16^ Many such historical descriptions are clouded by stigmatizing attitudes to migraine, FND and the women who most commonly suffer from them.

For much of the 20th century, migraine was, like FND, considered ‘psychosomatic’. For example, in 1970 Oliver Sacks wrote that ‘*A proportion of patients (perhaps an especially high proportion among sufferers from habitual migraine) and a number of physicians doubt or deny that migraine can be a psychosomatic illness and commit themselves to an endless search for physiological aetiologies and pharmacological treatments*’.^17^

The development of a neurobiological approach to migraine over the last 50 years is well-known.^2^ In the last two decades, there has been a renewed scientific interest in FND with an emergent non-dualistic model that incorporates neuroscience approaches but also psychosocial risk factors. There is increasing interest in neurological and psychiatric comorbidities of migraine,^18^ but thus far only very limited discussion of the overlap and interaction between two of the most common neurological conditions.^6^

### The frequency of motor and sensory FND co-occurring with migraine

#### The frequency of migraine in people with FND

We found nine studies reporting the frequency of migraine in people with FND ranging from 6 to 53% (Table 1). This data needs to be assessed alongside the frequency of migraine in reference populations in the context of the over-representation of females in FND samples; for example, the annual prevalence of migraine in one UK population sample was 7.6% of men and 18.3% of women.^28^ Only two studies reported the frequency of migraine using the International Classification of Headache Disorders criteria,^2,19,27^ whereas others relied on a ‘yes/no’ categorization from the records, the treating neurologist or the patient's self-report. Only three studies had migraine as a mandatory data point that would require a ‘yes/no’ response, whereas others recorded it as comorbidity when present so might have not identified all cases with migraine. Four were case–control studies comparing FND with epilepsy—although it should be noted that epilepsy is itself comorbid with migraine in excess of the population.^29^ In the three largest studies, migraine was found to be more common in individuals with functional seizures when compared to those with epilepsy, with odds of 2.6, 3.0 and 3.3^22,24,25^ in studies of 605, 282 and 13 640 patients, respectively. However, one smaller study of 170 patients reported no difference with odds of 1.0^23^

**Table 1:** Frequency of migraine in people with FND

First author	Setting, location	Study design^a^	FND patients			Controls			OR	Migraine diagnosis
			Subtype	*n*	Frequency of migraine	Type	*n*	Frequency of migraine		
Stone^19^	Neurology, Scotland	CC, P, M	Functional motor	107	36%	Other neurology and healthy	84	31%	OR 1.2	IHS criteria
Tinazzi^20^	Multicentre Neurology Cohort Italy	C, P, NM	Functional motor	410	5.8%	n/a	n/a	n/a	n/a	Notes review
Ducroizet^21^	Online, international	C (online), P, NM	Mixed^c^	524	13%^b^	n/a	n/a	n/a	n/a	Self-report
Elliott^22^	Epilepsy monitoring unit, USA	CC, P, NM	Functional seizures	324	53%	Epilepsy	281	30%	OR 2.6	Notes review
Gazzola^23^	Epilepsy monitoring unit, USA	CC, R, M	Functional seizures	85	8%	Epilepsy	85	7%	OR 1.0	‘Headache/migraine’
Kerr^24^	Epilepsy monitoring unit, USA	CC, R and P, M	Functional seizures	51	23%	Epilepsy	331	8.0%	OR 3.3	Notes review
Luthy^25^	Paediatric inpatients US	CC, R, NM	Functional seizures	399	17%	Epilepsy	13 241	6.4%	OR 3.0	‘Headache and migraine’
Massot-Tarrus^26^	Epilepsy monitoring unit, Canada	C, R, NM	Functional seizures	109	22%	n/a	n/a	n/a	n/a	Notes review
Riva^27^	Functional Movement Disorders clinic, Spain	C,P, M	Functional motor	51	45%	n/a	n/a	n/a	n/a	IHS criteria

^a^C, cohort; CC, case–control; P, prospective; R, retrospective; M, mandatory data item for migraine; NM, non-Mandatory data item for migraine.

^b^Forty-four reported migraine pre-FND diagnosis, 16 after and 8 reported undiagnosed migraine. IHS, International Headache Society.

^c^Self-report FND diagnosis.

In an international online study of 456 FND patients with mixed symptoms, 13% reported having migraines before their FND diagnosis.^21^ A number of other studies have reported the frequency of ‘headache’ in people with functional seizures^30^ or the frequency of headache combined with other pain symptoms,^31,32^ generally at a higher level than would be expected in the population.

One study reported a case series of 42 individuals with both functional seizures and headache, noting that 65% had migraine, with 73% reporting reductions in both functional seizures and headache during migraine treatment.^7^ Another prospective study of 51 individuals with functional movement disorders found that 40 (78%) had headache, or whom 23 met diagnostic criteria for migraine.^27^

There is therefore some evidence that migraine is more common in people with functional seizures than epilepsy, but data from functional motor symptoms and wider FND groups are less clear. Overall, the limitations of these data mean that it is not possible to conclude whether migraine is more common in FND than expected by chance.

#### The frequency of FND in people with migraine

We found four studies that addressed this question (Table 2). A 14-month prospective study in 1200 young adults reported roughly twice as many FND (‘conversion’) symptoms, approximately two per individual, in migraineurs compared to non-migraineurs.^33^ However, the nature of these symptoms and how they were identified was not specified.

**Table 2:** Frequency of FND in people with migraine

First author	Setting, location	Study design^a^	Migraine patients			Controls			Notes
			Subtype	*n*	Frequency of FND	Type	*n*	Frequency of FND	
Breslau^33^	Population, USA	P, CC, M,	Migraine	128	Mean 2.1 ‘conversion’ symptoms per patient	Healthy	879	Mean 1.1 ‘conversion’ symptoms per patient	*P* < 0.001; IHS criteria; nature of conversion symptoms unspecified
Chakravarty^34^	Neurology outpatients, India	P, C, M	Migraine (female)	656	23% functional seizures	n/a	n/a	n/a	IHS criteria; functional seizures were ‘swooning dissociative attacks’—diagnosed on clinical grounds, including 62 videos and 38 video EEG
			Migraine (male)	144	1.3% functional seizures				
			Migraine (paediatric)	200	5.5% functional seizures				
Youssef^35^	Paediatric Neurology outpatients, USA	R, C, M	Migraine (paediatric)	897	4.3% (chronic), 0.9% (episodic)	n/a	n/a	n/a	FND symptoms, including ‘nonepileptic shaking spells’, tremor and functional gait disorders
García-Albea^36^	Headache clinic, Spain	R, C, M	Migraine (inc. basilar)	1650	43/1650	n/a	n/a	n/a	Functional movement disorders 90%, seizures 37%, speech 35%

^a^C, cohort; CC, case–control; P, prospective; R, retrospective; M, FND mandatory data item; NM, FND non-mandatory data item.

In a single-centre study of 1000 migraineurs in India (800 adults and 200 children), functional seizures were found in 16.5% (19.3% of adults and 5.5% of children). All children and the vast majority of adults (152 of 154) with functional seizures were female. The majority were diagnosed only with semiology (so without EEG during habitual seizure), and all patients had ‘swoon type’ seizures. This high frequency of functional seizures, with high homogeneity of the seizure semiology, likely reflects local socio-cultural factors influencing both symptom generation and clinical presentation and has not been replicated in another study.^34^

A further study of 897 children with migraine described FND symptoms, including ‘nonepileptic shaking spells’, tremor and functional gait disorders in 4.3% of chronic migraine patients, and in 0.9% of those with episodic migraine. This was mostly during the migraine attacks, and many improved with migraine treatment.^35^ A frequency of 4.3% is higher than the population prevalence of FND, suggesting an elevated risk. This is perhaps the most persuasive study for an increased risk of FND in migraine, but there was little description of the FND symptoms themselves.

A study of 1650 tertiary headache clinic migraine patients described 43 with associated ‘conversion symptoms’, diagnosed with DSM-IV criteria.^36^ In total, 38 of the 43 individuals fulfilled criteria for basilar migraine. Some of the basilar migraine clinical features described also had features seen in FND, such as tubular vision, monocular diplopia and amnesia, which raises the question of whether these were better described as comorbid FND symptoms rather than basilar migraine (discussed further below). Neurological symptoms occurred before, during and after the headache in these patients. An additional report describes four patients with basilar migraine who had a positive diagnosis of FND, suggesting this is a phenotype where the intersection of FND and migraine should be especially studied,^37^ although there are no reports of an excess of FND symptoms in other case series of basilar migraine.^38^

Further studies have found higher frequencies of ‘somatoform dissociation’ in people with migraine versus controls^39^ and severe migraine versus less severe migraine,^40^ but these questionnaire-based studies cannot give an accurate estimate of FND diagnoses, especially since migraine itself is associated with many other physical symptoms aside from headache.

In conclusion, the published data is sparse and generally of low quality, and therefore, the prevalence of FND in people with migraine versus the general population remains uncertain.

#### Clinical overlap of FND and migraine

##### Migraine as a trigger for FND

Another potential relationship between migraine and FND is that migraine can, in some instances, be a precipitating event for the first episode of FND or a triggering event that leads to relapse of FND once it is established. Indeed, it would be surprising if it was not, since it is well established that any kind of medical condition, or even any symptom experience, can be sufficient to act as a trigger for a functional disorder.^3^ For example, persistent postural perceptual dizziness is typically precipitated by another vestibular disorder,^4,5^ functional seizures are much more common in people with epilepsy than the general population^41^ and irritable bowel syndrome is more than four times more likely to occur after a benign gastrointestinal infection.^42^ In a similar vein, if there was evidence that focal neurological symptoms arising during migraine aura were persisting longer than might be expected from an established or well-accepted migraine mechanism alone, then FND could be one potential explanation for this.

Several studies suggest that FND, or functional neurological symptoms, can be triggered by migraine. In 2006, Young *et al.* described what they termed migraine with unilateral motor symptoms (MUMSs) in 24 patients who were compared with 48 matched controls with migraine but no motor symptoms.^43^ Motor symptoms typically began with the onset of pain or worsened as the pain intensified. In 33%, weakness lasted 1–7 days and in 8% >7 days; 58% reported persistent weakness between headache episodes. Two patients with ‘inconsistencies on examination’ were excluded, but all 24 patients had ‘give-way’ weakness in more than one movement, a clinical feature of functional limb weakness.^44^ In total, 38% of patients with MUMS were told they had a stroke, and 17% believed they had had a stroke despite normal brain imaging. The authors concluded that MUMS could be caused by several issues, including prolonged aura or FND, but favoured the idea that the weakness was due to a ‘disordered protective reflex similar to that which causes give-way weakness in an injured limb and which is related to the severe allodynia that accompanies MUMS’.^43^ They noted the similarity to give-way weakness in complex regional pain syndrome, although that too is identical to the pattern seen in FND.^45^

Four other studies have looked retrospectively at precipitating events for functional motor disorders and found that migraine was an apparent trigger for some patients with motor symptoms lasting months and years, although the data were mixed. Migraine was noted as a precipitating factor for 3 out of 50 people with functional movement disorder (6%)^46^ and in 6 out of 49 (12%) patients with acute functional limb weakness.^47^ Another study examining triggers of exacerbations of functional motor symptoms noted only 3 out of 100 (3%) patients recorded headache as a trigger.^48^

A further UK study of patients diagnosed as having FND as a stroke mimic admitted to a hyperacute stroke unit noted a statistically significant increased presence of a history of migraine in 12% of 98 ‘functional’ mimics versus 5% in 163 ‘other medical’ mimics.^49^ In this group, about 18% of functional disorder cases had previously been given a diagnosis of ‘migraine’ or ‘hemiplegic migraine’. This study methodology could be criticized for over-ascertaining FND and including patients that other clinicians might have described as having migraine with aura.

##### The overlap of migraine aura and FND sensorimotor symptoms

In order to critically appraise these studies, it is important to consider the clinical nature and duration of sensorimotor symptoms in migraine aura. The most commonly reported reversible sensory symptom by people with migraine with typical aura is perioral and distal upper limb (‘cheiro-oral’) paresthesias. However, sensory loss may sometimes be experienced or articulated as weakness and/or heaviness of the limbs. We are not aware of any studies that clearly describe the clinical pattern(s) of weakness in migraine aura. For example, in hemiplegic migraine, is there a pyramidal distribution of weakness, with accompanying tone and reflex changes as seen in an upper motor neuron lesion, or is there a different clinical pattern such as a global distribution of weakness with a collapsing quality and dissociation between voluntary and automatic movements as seen in FND? From first principles, if one considers migraine aura as a dysfunction of cortical sensory processing secondary to cortical spreading depression in which there are proprioceptive and somatosensory changes, the pattern could be the same as in functional limb weakness. J Michell Clarke, in his original 1910 description of familial hemiplegic migraine, wrote, ‘*It is impossible not to be struck with the similarity of the hemiplegia… to the hemiplegia of functional disease, especially hysteria*’.^50^

The duration of motor aura in genetically confirmed cases of migraine is also relevant when considering whether the origin of prolonged symptoms after migraine may sometimes relate to an FND mechanism. A study of 208 patients with genetically confirmed hemiplegic migraine, migraine with aura that includes motor weakness, as well as 73 non-genetically confirmed cases, found that limb weakness duration was between 20 min and 24 h in all cases.^51^ This study also suggested that hemiplegic migraine is rarely associated with motor weakness only; almost all have at least two fully reversible other aura symptoms, such as visual, sensory or language disturbance.^51^ Other studies have reported longer durations, for example, 6 out of 89 patients in the first paper describing the CACNA1A genetic variant had auras lasting ‘up to 5 days’,^52^ with only one reporting an aura less than 24 h long. In other studies, 8 out of 65 had motor symptoms for longer than 24 h,^53^ and 4 out of 49 childhood-onset cases with PRRT2 gene variants had auras lasting more than 24 h, generally described as up to ‘several days’, with the longest specified as up to 10 days.^54^ The published data seems to indicate that in the vast majority of cases, motor aura symptoms resolve within 24 h, but can last for a few days and very rarely beyond 5 days.

The literature suggests that genetically confirmed patients with longer attacks can have abnormal neurological signs, almost always cerebellar, between attacks, and rarely these can be permanent. MRI head/EEG abnormalities and other signs of encephalopathy-like coma, fever and epileptic seizures can also occur. There are rare reports of similar persistent deficits in individuals with sporadic hemiplegic migraine.^55,56^ It is possible that prolonged aura is a consequence of repeated cortical spreading depression and/or prolonged recovery from hyperpolarization and/or the functional consequences of prolonged cerebral oligemia. Cerebral oligemia is documented as part of migraine with and without aura and has been shown to occur well into the postdromal phase of the attack.^57^ However, we have not been able to find reports of any patients with prolonged isolated hemiplegia considered solely due to migraine aura.^58^

Of course, there are several reasons why neurological symptoms may persist after migraine, and FND is only one of them. Migraine may also occur during a focal seizure with persistent weakness, or in fact during any focal neurologic disturbance of brain function that may sometimes be longer lasting, including stroke-like migraine attacks after radiotherapy (SMART attacks), ischaemic stroke and amyloid spells. Headache with neurological deficits and CSF lymphocytosis (HaNDL) is another clinical entity, which overlaps with hemiplegic migraine that may cause apparent prolonged aura (minutes to a few days, most a few hours), typically in association with CSF pleocytosis, MRI and EEG changes.^59^

##### FND as a trigger for migraine

Migraine and headache are also described as a ‘consequence’ of FND, especially functional seizures. A recent case series of 43 patients with functional seizures and migraine found that 28 individuals had migraine aura that interacted with symptoms before, during and after seizure episodes. Post-ictal headache has been reported as more common in epilepsy than functional seizures, but has rarely been studied.^60^ Anecdotally, we have often met patients with functional seizures who appear to have migraine triggered by their events.

In summary, there appears to be a variety of ways in which FND and migraine may overlap clinically. Migraine may trigger FND in some people, and it is possible that the transient acute motor and sensory symptoms and signs of migraine may overlap with those seen in persistent FND, with uncertainties about their clinical characteristics and duration.

## Shared demographics and risk factors for migraine and FND

If migraine and FND potentially share some mechanistic overlap, then you would also expect them to share some demographic features and risk factors. In this section, we examine the evidence for similarities in sex ratio, genetics and environmental risk factors such as stress/trauma and comorbidities. See Fig. 1 for a summary of these similarities.

### Female preponderance and age of onset

Female preponderance is notable in both migraine^2^ and FND, both being around three times more common in women.^3^ The age distribution of the highest prevalence and age of onset also shows similarities,^61,62^ with a rapid rise in incidence through the teenage years, a peak in the twenties and thirties and then a subsequent decline. However, many other conditions, such as multiple sclerosis and many autoimmune conditions, are also similarly more common in women and have similar distributions of age of onset, so this similarity provides little, if any, support for mechanistic overlap.^63^

### Genetic risk factors

Migraine is moderately heritable, with estimates of heritability up to 60%. Genetic research in FND is in its infancy,^64,65^ and there have been no studies of heritability. FND has also been reported in more than one family member.^66^ Other disorders that are risk factors for FND, such as anxiety and depression, post-traumatic stress disorder, obsessional personality traits, autism, fibromyalgia and hypermobility spectrum disorder, all have high heritability based on twin studies and other genetic studies. It would be surprising, but not impossible, if FND did not have some heritability in this context. There may be shared genetic risk factors between migraine and FND, but there is no current evidence to link them on this basis.

### Stressful life events and trauma

#### FND

Stressful life events and trauma, both around symptom onset and in earlier life, especially childhood, have historically been emphasized as a key risk factor in the development of FND. In fact, this risk factor assumed the status of ‘the cause’ of the disorder for most of the 20th century, enshrined in the diagnostic label ‘conversion disorder’ reflecting the Freudian view that psychological stress was ‘converted’ into physical symptoms. However, this view has significantly shifted in recent decades as significant stressors are not found in all FND patients, even when looked for with optimal methodology, and even when they are found, establishing causality is challenging. Severe stressors and trauma are common in all populations^67^ and associated with a wide range of physical as well as psychiatric conditions, including migraine.^68^ In addition, there is interesting evidence that retrospective assessment of childhood trauma has poor agreement with prospective assessment.^69^ As an example, one study of children with documented maltreatment found a much lower frequency of mental and physical health conditions than expected,^70^ suggesting that in some cases, measurement of adversity could be interpreted as measurement of perception of adversity.,

In a systematic review of 34 case–control studies, 1405 people with FND demonstrated an increased frequency of childhood and adulthood stressors in FND compared with controls. The odds ratios (ORs) were higher in people with FND for emotional neglect in childhood at 5.6, childhood sexual abuse 3.3 and physical abuse 3.9. Nevertheless, included studies also demonstrated that many people with FND (anything from 15 to 70%) do ‘not’ have recorded severe adverse life events.^71^ Recent stressful life events were less strongly associated with FND in this meta-analysis (OR 2.0–2.5 versus neurological or healthy controls), although there is evidence of escalation of risk just before the onset in some individuals. Modern research therefore places stress and adverse experience as one of many potential risk factors for FND, which includes other events such as physical injury, pain or having other neurological conditions, including migraine, as mentioned previously.

#### Migraine

A relationship between retrospective reports of stressful life events and migraine is well established. A meta-analysis of 28 studies involving over 150 000 participants found a pooled OR of 1.48 for one or more adverse childhood experiences, rising to an OR of 2.1 for ≥4 adverse experiences. Threatening adverse experiences seemed to predict the presence of migraine independently compared to neglectful experiences, suggesting potentially different pathways to vulnerability.^72^ In the case of emotional abuse, this association is maintained independently of comorbid depression and/or anxiety. One study found that individuals with migraine who had adverse experiences were more likely to have vascular biomarkers possibly related to early life stress, causing endothelial activation and then migraine.^73^ A caution here is that ‘neuroticism’ is a risk factor for migraine and may also be a risk factor for retrospective recall of childhood adversity.^69^

Stress, and the relief of stress, as seen in ‘leisure migraine’, is an established migraine trigger. Evidence from prospective studies suggests it may be especially relevant when episodic migraine becomes chronic,^74^ and there are notable reports of migraine occurring more frequently than expected by chance after a stressful event, for example, the tragic mass shooting of children in Norway in 2011^75^

In conclusion, the frequency of stressful life events is increased in both migraine and FND, particularly for FND, but this is also the case for many other neurological disorders, as well as most psychiatric and physical health disorders.

### Psychiatric comorbidity

#### FND

Studies have demonstrated high frequencies of common psychiatric comorbidities, including depression, anxiety and panic disorder in people with FND.^76^ Post-traumatic stress disorder (PTSD) and complex PTSD are also commonly diagnosed, as would be expected given the previously mentioned elevated rates of psychological trauma. Case–control studies demonstrate higher comorbidity compared to patients with similar neurological symptoms, such as epilepsy or Parkinson's disease, as well as healthy controls.^77^ These have not yet been synthesized in a format that allows a clear description of ORs. Cross-sectional studies typically find frequencies of 30–50% for depression and anxiety and 20–30% for PTSD.^77,78^

#### Migraine

Migraine has been associated with an increased risk of depression (two to three times more common), anxiety (two to five times more common), obsessive compulsive disorder and PTSD.^79,80^ PTSD is comorbid with migraine at a frequency of 14–25% (versus 1–12% population controls); it is more prevalent in patients with chronic migraine compared to episodic migraine (43% versus 9%). This difference does not seem to be explained by a difference in the frequency of trauma exposure between the groups,^81^ and PTSD may mediate the relationship between past psychological trauma and migraine described above.

In conclusion, psychiatric disorders are more common in both migraine and FND than in the general population, but they are also significantly higher in most neurological conditions such as epilepsy,^82^ multiple sclerosis^83^ and Parkinson's disease.^84^ It is therefore hard to draw any epidemiological conclusions regarding the relationship between the two disorders in relation to this comorbidity.

### Functional disorder comorbidity

A considerable amount of epidemiological evidence indicates substantial overlap between common functional (somatic) disorders, including irritable bowel syndrome, persistent fatigue states and chronic primary pain syndromes such as fibromyalgia.^85^ A similar overlap for both FND and migraine would support a mechanistic connection.

#### FND

Other functional disorders are more common in people with FND than in the general population. A recent meta-analysis (*n* = 4272) found that an estimated 55% of people with FND have chronic pain, 16% irritable bowel syndrome (IBS) and 10% fibromyalgia (all higher than the control population). In case–control studies, pain has been recorded in FND at roughly twice the rates of other neurological disorders, such as epilepsy.^86^

#### Migraine

The literature in migraine suggests a similar increase in vulnerability to functional disorders.^85^ In a meta-analysis of 4.2 million individuals with primary headache, at least 82% of whom had migraine and 26% had fibromyalgia, around three to six times more than the population estimates of between 2 and 8%.^79,87^ A further German insurance data study of 56 597 adolescents found that those with migraine had a higher risk of developing ‘back pain’ (OR 1.6) and irritable bowel syndrome (OR 1.5) during a 10-year follow-up period.^88^ A multicentre US and Canada study of 1348 people with migraine found fibromyalgia in 10%. A Swedish study found irritable bowel syndrome in 7% and chronic fatigue syndrome in 3% of 151 migraine patients with ORs of between 3 and 6 compared to 3255 controls^89^. A retrospective registry-based prevalence study in Taiwan found that 6902 migraine patients were at a 1.5-fold increased risk of developing chronic fatigue syndrome compared to 27 608 controls.^90^ The same database also found a 1.9-fold increased risk of irritable bowel syndrome in 14 117 migraine patients compared to controls, a relationship that was especially strong in people under 30.^91^ A similar 2-fold increase has been seen in migraine in other studies of people with irritable bowel syndrome.^92^

Persistent postural perceptual dizziness (PPPD) was not included in our search strategy for this review, because its relationship to migraine has been explored in other literature, but is commonly regarded as a subtype of FND.^3^ Persistent postural perceptual dizziness is commonly triggered by migraine. In one study of 59 individuals with persistent postural perceptual dizziness, 53% met formal diagnostic criteria for migraine.^93^ In persistent postural perceptual dizziness, vestibular disorders, including vestibular migraine, not only trigger the condition but are also commonly comorbid as a perpetuating factor.

An increase in functional disorder comorbidity is also reported in other neurological conditions. For example, data from small cohorts suggests that people with epilepsy and multiple sclerosis also appear to have an increased frequency of irritable bowel syndrome and fibromyalgia compared to healthy controls, although data is limited and based on small cohorts.^94,95^ It is therefore not clear that migraine has a unique epidemiological relationship to functional disorders.

### Neurological disorder comorbidity

One of the ‘rediscovered’ features of FND in recent years has been the recognition of the importance of neurological comorbidity. The experience of neurological symptoms from another cause appears in many individuals to be an important predisposing, precipitating and perpetuating factor for FND. This association has been shown best in epilepsy, where up to 5% of patients may have functional seizures,^41^ Parkinson's disease that can trigger FND, especially in the prodromal period,^96^ and multiple sclerosis.^97^ Migraine is also more common in other neurological disorders, including epilepsy and stroke.^18^

## Shared pathophysiological mechanisms between FND and migraine

Current ideas about the mechanisms of FND and migraine overlap, especially with respect to the concept of allostatic load and predictive processing models of brain function.

Allostasis refers to the adaptive regulation of brain and body physiology based on current and predicted outcomes related to internal sensations and external environments. In essence, it is an adaptation of the concept of homeostasis but with feedforward mechanisms included in the model. Abnormal allostatic load refers to persistently high ‘input’ that leads to maladaptive or dysregulated responses. This is compatible with previous ‘stress diathesis’ models, whereby a threshold for stress to cause symptoms is reached by varying combinations of risk factors, including the level of environmental stress.^68^

Predictive processing models of brain function have been applied to numerous clinical brain conditions, including FND, chronic pain, schizophrenia and phantom limb syndrome.^98^ In all these disorders, the theory posits a model in which the ‘top-down’ model of what the brain expects ‘overrides’ the ‘bottom-up’ sensory input, which otherwise might correct it. In phantom limb syndrome, for example, the prediction of the presence of a limb overrides competing sensory information that it has been amputated. In functional limb weakness and sensory loss, the prediction of reduced movement or sensation is hypothesized to override sensory information indicating intact limb anatomy and physiology. An individual is therefore unable to accurately appraise or ‘reset’ their experience, and therefore symptoms persist. For transient forms of FND, such as seizures and paralysis, internally generated predictions of symptoms can be seen as an adaptive way to deal with autonomic arousal and fear and pain. For persistent FND symptoms, however, the symptoms are learnt in the absence of a stimulus corresponding to overweighted predictions and are maladaptive. More advanced models in FND suggest that altered prediction errors of interoception and emotion category construction in the context of allostatic load may be important components in the generation of symptoms.^98^

Allostasis and predictive processing have also been recently integrated into a mechanistic account of migraine,^99,100^ which proposes that symptoms arise from multimodal sensory amplifications of prediction errors initiated as forms of a ‘failsafe’ procedure to maintain allostasis. Put more simply, the brain shuts itself down, in anticipation or as a consequence of being overloaded, so it can ‘reset’.^100^ In chronic migraine, there may be a similar ‘learnt’ component corresponding to overweighted predictions.

In both disorders, therefore, it has been proposed that there is abnormal prediction error learning in response to allostatic load. In both disorders, there is also a response that could be seen as initially adaptive but is repeated or prolonged and therefore becomes maladaptive. In the case of migraine, this response shuts down the brain with headache, immobility and avoidance of sensory input. In the case of FND, there is compartmentalization and shutting down of a body part leading to limb weakness or numbness, or in the case of functional seizures, shutting down to the point of potentially losing control of the whole body and even awareness. We recognize that predictive processing and allostasis are broad frameworks that have been applied to many neurological and psychiatric disorders, and that while conceptually appealing, they remain one of many competing frameworks that still require more substantiation. Nonetheless, such frameworks are directly applicable to treatment, for example, to rehabilitation approaches to both conditions.

Persistent postural perceptual dizziness, often considered a subtype of FND, is often triggered by migraine, especially vestibular migraine. Authors exploring this overlap have suggested that, over and above this, individuals with migraine are particularly likely to experience persistent postural perceptual dizziness because of shared differences such as lowered perceptual thresholds for motion perception and an increase in visual motion sensitivity.^4^

Functional neuroimaging, neurophysiology and experimental psychology studies show some potential overlap in processes of disordered attention, sensory processing, interoception, emotional processing, agency, dissociation and other putative mechanisms. For example, patients with migraine have reduced habituation, suggesting a deregulation of cortical excitability, for example, with median nerve somatosensory evoked cortical recording.^101^ This is similar to findings of impaired sensory attenuation in motor FND^102^ and during a forced matching task.^103^ Delayed habituation of learning was also found in functional movement disorder using a broken escalator paradigm.^104^ Impaired prepulse inhibition of the R2 response of the blink reflex in functional movement disorder compared to controls^105^ suggests altered processing or ‘gating’ of somatosensory inputs at a brainstem level, something that has also been seen in migraine^106^ and in fibromyalgia.^105^ This parallels a finding of impaired descending conditioned pain modulation, measured by pain-evoked sensory potentials (N2/P2 complex) in individuals with functional movement disorder,^107^ which has also been a finding in migraine.^108^

Studies using functional MRI and transcranial magnetic stimulation in both disorders have revealed potential commonalities but also differences in brain activations.^57,109^ Functional neuroimaging of FND and migraine has noted the activation of the salience network (in migraine, especially when symptoms are occurring), as well as abnormal limbic system connectivity. In contrast, studies in migraine have not highlighted network changes in relation to the sense of agency that is core to many FND experiences, such as hypoactivity of the right temporo-parietal junction. Migraine functional neuroimaging often highlights brainstem activation, which is less common in FND studies, although activation of periaqueductal gray has been a feature in a number of studies in FND,^110^ and is of interest because of its role in freeze/fear response networks.

Overall, different paradigms and experimental approaches limit current comparison or meaningful synthesis, but we would encourage further investigation like this. Treatment for migraine has advanced by studying concrete neurophysiological and testable scientific hypotheses. Similar evolving neurophysiological, genetic, neuroimaging and neurotransmitter research in FND could lead to similar advances in that disorder. The scientific field does not have to choose between ‘top-down’ predictive processing models and ‘bottom-up’ neurophysiological perspectives, since both are likely to be relevant in FND and in migraine.

## Summary of evidence, clinical and research implications

### Summary of evidence

The data regarding the frequency of FND in migraine and vice versa suggests that migraine may be more common in people with functional seizures than those with epilepsy, but the quality of the data is too low to draw firm conclusions beyond this. The literature allows us to conclude that FND can be triggered by episodes of migraine, especially those with aura. However, it is not clear whether migraine is especially likely to trigger FND compared to other neurological conditions or physical experiences. Migraine is the most common cause of neurological symptoms in the general population, so one might expect it to be one of the commonest triggering factors for FND as well. The frequency, duration and clinical features of persistent motor and sensory symptoms arising in the context of migraine aura deserve more study.

None of the available data on shared demographics or risk factors allows a clear link to be made between FND and migraine. All the risk factors discussed may be confounded by referral and diagnostic bias—for example, people who experience life stressors/trauma have psychiatric disorders, or other functional disorders (including FND) may be more likely to be referred and diagnosed. In addition, living with any chronic condition likely increases the frequency of psychiatric and functional disorders, and here a comparison with other neurological conditions such as epilepsy and multiple sclerosis is especially salutary. Migraine and FND do share contemporary hypotheses regarding aetiology and mechanism, but there is insufficient evidence to conclude more about their relationship at the current time.

### Clinical implications of the overlap between FND and migraine

There are several potentially beneficial reasons to look more closely at the overlap between FND and migraine from a purely clinical perspective. Individuals with prolonged weakness after migraine aura, especially for weeks rather than days, who have a pattern of weakness similar to FND, may benefit from new FND-focused approaches to physiotherapy.^111^ Randomized trials of people with functional motor disorders of an average duration of 5 years have shown benefit, either from clinical global outcome measures^112^ or in activities of daily living,^113^ from an intensive physiotherapy treatment that capitalized on some of the unique features of functional motor disorders, including improvement with distraction. The authors of this paper have met many patients labelled as having ‘hemiplegic migraine’ where ultimately a reformulation of their condition as functional neurological symptoms or FND proved more useful in terms of treatment and improvement of symptoms.

People with FND often have many different symptoms and difficulties to assess, and migraine can often get overlooked if not prioritized and specifically enquired about.^114^ Prodromal and postdromal symptoms of migraine, such as fatigue and impaired cognition, need to be teased out from other causes of similar symptoms, as they may benefit from specific migraine therapy. Recognition and improvement of migraine treatment in our experience can also sometimes improve FND symptoms when present.^114^ It is also our experience that the severity of FND can influence the severity of migraine and vice versa. We do not know whether newer treatments for migraine based on calcitonin gene-related peptide and other mechanisms may have specific benefits in FND or other functional disorders with pain, such as fibromyalgia and irritable bowel syndrome.

### A research agenda investigating the overlap between FND and migraine

This narrative review highlights significant gaps in our understanding of the overlap between FND and migraine. The epidemiological studies to date are generally of low quality, and most did not use standardized migraine or FND diagnostic criteria.

Better quality studies of the overlap are needed to determine the true frequency of migraine in FND, especially of FND in migraine populations. At present, we do not appear to know whether the pattern of weakness in migraine aura is the same in both genetic hemiplegic migraine and sporadic migraine aura and whether it approximates more to pyramidal weakness as seen in stroke or global collapsing weakness as seen in FND. We do not understand what the maximum duration of persistent migraine aura is, and when, on that basis alone, it becomes unreasonable to attribute that purely to migraine pathophysiology.

At a broad pathophysiological and aetiological level, FND and migraine are both disorders of abnormal nervous system functioning that are influenced, like many brain disorders, by stress and the environment. The stigmatized history of migraine is perhaps one reason why there has been, at least in the last few decades, some reluctance to study its psychosocial risk factors, which are now increasingly recognized.^9^ Simultaneously, FND, which had been saddled with a purely psychosocial explanation for nearly a century, has seen an increase in research exploring its pathophysiological mechanisms, which are now beginning to show some convergence with that of migraine.

Studying the relationship between FND and migraine requires putting aside dualistic preconceptions about the boundaries and mechanisms of both disorders to determine what each can learn from the other, to what extent these common and often highly disabling conditions are related and how they interact with one another (Box 2).

Box 2 Research agenda

- Epidemiology: High-quality studies of comorbidity using established diagnostic criteria as close as possible to population samples. There are particular limitations of current diagnostic criteria for FND concerning asymptomatic FND motor and sensory signs and defining thresholds for a disorder versus a symptom.^115^
- Clinical phenotyping: Detailed and thorough clinical studies, particularly in subtypes where there are currently clinical challenges, uncertainties and a lack of consensus (e.g. clinical features and boundaries of atypical hemiplegic migraine without clear family history and/or genetic abnormality).
- Mechanistic studies: Focusing on exploring areas of potential mechanistic overlap informed by predictive processing and allostasis models of both disorders, the proximal causes and mechanisms of potentially shared clinical features such as give-way weakness and commonalities in other neurophysiological and functional neuroimaging studies.
- Greater collaboration: Enhanced crosstalk, training and co-working on planning and executing research between migraine and FND experts. This should be at all levels, from clinical assessment, with greater awareness and clinical training in both disorders, to research planning and efforts on the above topics.

## Data Availability

Data sharing is not applicable to this article as no new data were created or analysed in this study.
